# Evaluation of math anxiety and its remediation through a digital training program in mathematics for first and second graders

**DOI:** 10.1002/brb3.2557

**Published:** 2022-03-29

**Authors:** Chan‐Tat Ng, Yin‐Hua Chen, Chao‐Jung Wu, Ting‐Ting Chang

**Affiliations:** ^1^ Department of Psychology National Chengchi University Taipei City Taiwan, ROC; ^2^ Research Center for Mind, Brain, and Learning National Chengchi University Taipei City Taiwan, ROC; ^3^ Graduate Institute of Athletics and Coaching Science National Taiwan Sport University Taoyuan City Taiwan, ROC; ^4^ Department of Education Psychology and Counseling National Taiwan Normal University Taipei City Taiwan, ROC

**Keywords:** cognitive training, computerization technology, exposure therapy, game‐based learning, math anxiety, mathematical learning

## Abstract

**Introduction:**

Math anxiety severely impacts individuals’ learning and future success. However, limited is understood about the profile in East Asian cultures where students genuinely show high‐level math anxiety, despite that they outperform their Western counterparts. Here, we investigate the relation between math anxiety and math achievement in children as young as first and second graders in Taiwan. Further, we evaluate whether intensive exposure to digital game‐based learning in mathematics could ameliorate math anxiety.

**Methods:**

The study first evaluated a group of 159 first and second graders’ math anxiety and its correlation with math performance. Subsequently, a quasi‐experimental design was adopted: 77 of the children continued and participated in multi‐component digital game training targeting enumeration, speeded calculation, and working memory. Post‐assessment was administered afterward for further evaluation of training‐associated effects.

**Results:**

Results confirmed that math anxiety was negatively associated with school math achievement, which assessed numerical knowledge and arithmetic calculation. Furthermore, children's math anxiety was remarkably reduced via digital training in mathematics after 6‐week intensive remediation. Crucially, this math anxiety relief was more prominent in those with high‐level math anxiety. Although the children who underwent the training showed training‐induced math achievement and working memory enhancement, this cognitive improvement appeared to be independent of the math anxiety relief.

**Conclusion:**

Our findings demonstrate that students can show highly negative emotions and perceptions toward learning even in high‐achieving countries. Auspiciously, the feeling of distress toward learning has the feasibility to be relieved from short‐term intensive training. Our study suggests a new approach of early treatments to emotional disturbance that can lead to permanent consequences in individuals.

## Graphical abstract

First and second graders show negative association between math anxiety and math performance. After 6‐week intensive remediation, children with high initial math anxiety showed remarkable reduce in math anxiety level. Furthermore, the worse their math anxiety is, the greater these children benefit from math anxiety remediation.

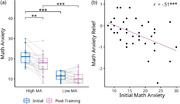

## 1 INTRODUCTION

Math anxiety is a severe problem pessimistically affecting individuals’ mathematical learning and achievement, as well as their academic and professional success by causing avoidance of mathematical activities (Ashcraft & Krause, 2007; Foley et al., 2017; Passolunghi, 2011). It is a specific negative emotional reaction accompanied by feelings of tension, apprehension, and fear under math‐related problem solving or learning situations (Ashcraft, 2002). Despite the severity, nearly half of global students have been reported to suffer from worrying about failure in math learning (OECD, 2013). Thus, both identification and remediation of math anxiety appear to be crucial for supporting mathematical learning as well as future school success. In this study, we investigate math anxiety of children as young as first and second graders in the East Asian culture where students usually suffer from excessive math anxiety, even though their math achievements are exceptional worldwide (Foley et al., 2017). Furthermore, we examine whether their math anxiety could be ameliorated through intensive exposure to a computer‐assisted training program that is designed based on the guidepost of the theoretical framework of how math anxiety impacts math achievement.

### 1.1 Negative association between math anxiety and math performance

Math anxiety has been extensively identified as having negative impacts on math performance (Ashcraft, 2002; Ashcraft & Kirk, 2001; Maloney & Beilock, 2012; Ramirez et al., 2018; Wu et al., 2012). As reviewed by Ramirez et al. (2018), there are two main accounts interpreting the adverse effects of math anxiety. One of which is the disruption account, which believes that the impact of math anxiety on mathematical learning originates from limited working memory (Ashcraft & Kirk, 2001; Ramirez et al., 2013). Ashcraft and Kirk (2001) reported that college students with high math anxiety were significantly less accurate in performing addition problems with carry operation only when a secondary task that required a high working memory load was conducted. In another study, Ramirez et al. (2013) reported that the negative association between math anxiety and math performance was more prominent in those with better working memory capacity. These results have led the authors to suggest that individuals with high working memory capacity have the tendency to rely on their talented working memory to solve math problems, and such strategies can be blocked when working memory is disrupted. Therefore, the impact of math anxiety on learning can possibly be due to the disturbance of working memory strategies while performing mathematical tasks.

An alternative interpretation is the competency account. This approach argues that math anxiety and poor math performance are the outcomes, rather than the origins, of poor math ability. One supportive study was conducted on undergraduate students by Maloney et al. (2010). In that study, participants with high math anxiety made more errors than their low‐anxiety peers when performing enumeration on quantity greater than five that requires counting but not on subitizing, that is, enumeration over quantity less than 5. This suggested that math anxiety can arise when the task is more demanding. According to the above two theories, it is recommended that interventions aiming to reduce math anxiety and its effects on performance should not only focus on numerical skills but also working memory.

### 1.2 Math anxiety in young children

Math anxiety can emerge and exhibit adverse impacts early and deteriorates across school stages (Ramirez et al., 2013; Wu et al., 2012; Young et al., 2012). In a behavioral assessment, Wu et al. (2012) reported negative correlations between the measured math anxiety level of second to third graders and their calculation as well as mathematical reasoning skills. Similar effects were found in younger children in first and second grades (Gunderson et al., 2017). Chiu and Henry (1990) reported a negative correlation between math anxiety and math grades, particularly in children above fifth grade. Beyond childhood, math anxiety can worsen over time (Hembree, 1990; Ma, 1999). Biatchford (1996) reported there were much fewer high‐school than elementary‐school students reporting mathematics as their favorite subjects. In a meta‐analysis study that primarily included adult participants, Hembree (1990) found that math anxiety was consistently associated with poor mathematical attainments. All these studies have reported moderate to strong effect sizes, with correlation coefficients ranging from −.24 to −.50. Altogether, these findings suggest that bearing negative emotions at the starting level may lead to lifelong detrimental effects on mathematical achievements.

### 1.3 Math anxiety in the East Asian culture

Culture is another significant factor that affects math anxiety. According to normative global surveys evaluating school‐level classroom performance in mathematics, East Asian countries and economies, such as Singapore, South Korea, Hong Kong, Taiwan, and Japan, usually outperform many of their global counterparts (Chang et al., 2019; Mullis et al., 2012, 2016; OECD, 2013). Paradoxically, these East Asian countries display tremendously low scores on students’ self‐reported liking for learning mathematics, high levels of worrying about failure in math performance (Mullis et al., 2012, 2016; OECD, 2013), and low math self‐concept as well as self‐efficacy (Lee, 2009). A more recent PISA assessment reveals that students in these Asian countries show high‐level fear of failure in general, with Taiwan expressing the greatest fear of failure among all participating countries and economies (OECD, 2019). These negative attitudes toward math and the overly low learning motivation in Asian students can be attributed to the parenting style, highly demanding learning environment (Chang et al., 2019), and the examination system (Tan & Yates, 2011). Using the meta‐analytic approach, Zhang et al. (2019) revealed that the negative correlation between math anxiety and math performance was stronger in studies involving Asian students than those involving European students. Together, these results have suggested that the crucial bottleneck of mathematical learning in Asian students is possibly the learning motivation rather than their actual performance or competence. Lacking self‐initiated incentives for learning may result in students’ reluctant feelings and unwillingness to study advanced mathematics. Nevertheless, still, limited attention has been received in systematically assessing math anxiety profiles in these East Asian students. The lack of knowledge for its remediation is even more concerning, especially in the early stages of education, as it can be crucial for children's future success.

### 1.4 Remediation of math anxiety

For treatments of anxiety disorders, exposure‐based cognitive tutoring is one potential strategy (Abramowitz et al., 2011). This technique asserts that repeated and intensive exposure to anxiety sources without the intention to cause any actual hazard potentially reduces negative emotions. Numerous studies have demonstrated its effectiveness in treating anxiety disorders such as post‐traumatic stress disorder (Van Etten & Taylor, 1998) and specific phobia (Wolitzky‐Taylor et al., 2008). Supekar et al. (2015) further adapted this method for remediating math anxiety. In their study, a group of third graders participated in an intensive one‐to‐one math tutoring program for 8 weeks. After the tutoring program, only children with high‐level math anxiety but not their low‐anxiety peers showed relief from such negative emotions. Further, these children demonstrated neural recovery from excessive activation and abnormal functional connectivity in the amygdala, the brain circuitry constantly associated with fear and anxiety. This study provides supporting evidence that math anxiety can be alleviated by short and sustained daily exposure to mathematical practice. Moreover, this study has provided the biological bases of math anxiety as well as the corresponding neuronal modulation through the practice of mathematical intervention. Likewise, Choe et al. (2019) revealed that mandatorily exposing participants to complex multiplication problems can suppress their math avoidance behaviors. Together, these studies have supported the idea that increased exposure to math‐relevant materials can be beneficial to learning for highly math‐anxious individuals. Therefore, the exposure‐based intervention method appears to be a promising approach to math anxiety remedy and further contributes to understanding treatments of emotion‐related learning disorders.

Relative to conventional classroom teaching and one‐to‐one tutoring, game‐based learning, referring to the learning process through gameplay, provides an interactive and playful environment for learning (Plass et al., 2015). One of the essential elements of game‐based learning is that it uses game components, such as incentive systems, to motivate learners to engage in tasks that may not be appealing (Plass et al., 2015). When implemented with computerization technologies, game‐based learning has further provided valuable insights to enhance learning through digital platforms (Butterworth & Laurillard, 2010; Räsänen et al., 2009). Besides providing a customizable, sharable, and motivating form of learning, computerization technologies allow easy tracking of students’ learning progress for both teachers and researchers (Butterworth & Laurillard, 2010; Räsänen et al., 2009).

Digital game‐based learning has been extensively implemented on mathematical intervention in school‐age children and low‐attaining learners (Butterworth & Laurillard, 2010; Butterworth et al., 2011; Nemmi et al., 2016; Räsänen et al., 2009; Sanchez‐Perez et al., 2017; Wilson, Dehaene, et al., 2006; Wilson, Revkin, et al., 2006). One pioneering digital training program, *Number Race*, was designed based on the training in magnitude representation precision (Wilson, Dehaene, et al., 2006; Wilson, Revkin, et al., 2006). In another example, *Graphogame‐Math* trained children to link small sets of objects to verbal labels of numbers (Räsänen et al., 2009). Both games were found effective in improving mathematical skills, including enumeration and calculation, particularly for those with mathematical learning difficulties (Räsänen et al., 2009; Wilson, Dehaene, et al., 2006; Wilson, Revkin, et al., 2006). Nemmi et al. (2016) further proposed a composite intervention program in which 6‐year‐old children were exposed daily to digital games for number line and working memory training for eight weeks. Rather than training in either number line or working memory alone, a combination of the two genres resulted in the most significant advantage for improving children's arithmetic problem‐solving skills (Nemmi et al., 2016). Sanchez‐Perez et al. (2017) trained children to play the n‐back task, span task, and calculation on different imaginary planets. They found that after 1‐h weekly training for 13 weeks, school attainments of mathematics were significantly improved in children between third and sixth grades (Sanchez‐Perez et al., 2017). Although the measurements of training effects greatly varied in these previous studies, they exhibited overall moderate to large effects, with reported partial eta squared of up to .19. These studies have provided pioneering empirical evidence that targeted interventions coupled with computerization technologies can be potential strategies for remediating the mathematical skills of both typical and low‐achieving students. It is intriguing to further examine whether intensive exposure to the targeted mathematical materials can, in the meantime, ameliorate emotion‐related learning difficulties.

It is argued that children's negative feeling associated with math is likely reduced in the learning environment using computerization technology (Sun & Pzydrowski, 2009). However, existing literature investigating this idea has yielded inconsistent results. In one study, Jansen et al. (2013) examined math anxiety changes of children in Grades 3 to 6 after they played an adaptive web‐based computer game, *Math Garden*. This game is designed to train arithmetic skills and can be freely played at school or at home. However, unlike the above‐reviewed study of Choe et al. (2019), children did not show math anxiety relief after playing the game for 3 to 6 weeks (Jansen et al., 2013). In a longitudinal study, Vanbecelaere et al. (2020) assessed the effect of a numerosity evaluation computer game on math anxiety levels for first graders. Although the overall math anxiety level decreased after the intervention, no difference was found between the experimental group and the control who received a regular math curriculum. One possibility of their results is that the program was conducted during school hours, and the game content was aligned to the regular curriculum. It was the difference between conventional classroom setting and game‐based environment, rather than math anxiety intervention, being compared. Altogether, these previous efforts have suggested that the training contents and materials shall be carefully deliberated.

### 1.5 Overview of the current study

We investigate math anxiety of young children in Taiwan, a country in the East Asian cultures with higher‐than‐average mathematics performance but elevated math anxiety in cross‐national assessments (Mullis et al., 2012, 2016; OECD, 2013). We focus on first and second graders in this study, as formal math curriculums are not introduced until this school stage. We first examine first and second graders’ math anxiety and its relationship with mathematical achievement. Based on the extensive Western literature suggesting that young children do suffer from math anxiety (Gunderson et al., 2017; Ramirez et al., 2013; Supekar et al., 2015; Wu et al., 2012; Young et al., 2012), and the situation deteriorates in East Asian countries (Mullis et al., 2012, 2016; OECD, 2013), we expect that math anxiety would start manifesting in Taiwanese first and second graders, and it should be negatively correlated with math school attainments.

Next, to investigate whether excessive math anxiety could be ameliorated, we implement a digital version of gamed‐based training and apply the exposure‐based cognitive training framework on first and second grade school children. Because previous findings suggest that composite training could yield better outcomes than single‐construct training (Nemmi et al., 2016), our training program is designed as a multifaceted approach that includes multiple training modules. According to the disruption account which emphasizes the comorbidity between math anxiety and working memory, our training program contains not only numerical skill modules but also a working memory unit (Nemmi et al., 2016; Obersteiner et al., 2013). As previous studies have shown that exposure‐based therapies can be adopted to ameliorate anxiety disorders (Abramowitz et al., 2011; Supekar et al., 2015), and learning motivation can be upregulated by the digital environment (Sun & Pzydrowski, 2009; Verkijika & De Wet, 2015), we predict that, coupled with intervention designed based on the cognitive characteristics of math anxiety, the digital program in this study would alleviate children's math anxiety. Particularly, according to Supekar et al. (2015), we expect the remediation effect to be more prominent in those who bear heavy burdens of emotion‐related learning problems.

Finally, we assess whether the digital game‐based training would improve school‐level mathematical achievements as well as working memory capacity. Because core math skills and working memory are included in the composite training, we hypothesize that children's math performance and working memory will be enhanced after training. We also predict that improvement in both math performance and working memory would be correlated with the remediation of math anxiety.

In sum, the current study focuses on testing the following three hypotheses:

**Hypothesis 1**: Children as young as first and second graders in Taiwan, an East Asian culture that shows a high‐pressure learning environment, would show high math anxiety, and this math‐related negative emotion would be negatively correlated with school performance in math.
**Hypothesis 2**: Prolonged exposure to digital game‐based learning that includes numerical and working memory modules could ameliorate children's math anxiety, especially for those who are high in math anxiety.
**Hypothesis 3**: Core numerical skills and working memory would be enhanced by the training, as these cognitive skills are included in the training modules, and the performance gain would be associated with math anxiety relief.

## 2 METHODS

### 2.1 Participants

**FIGURE 1:**
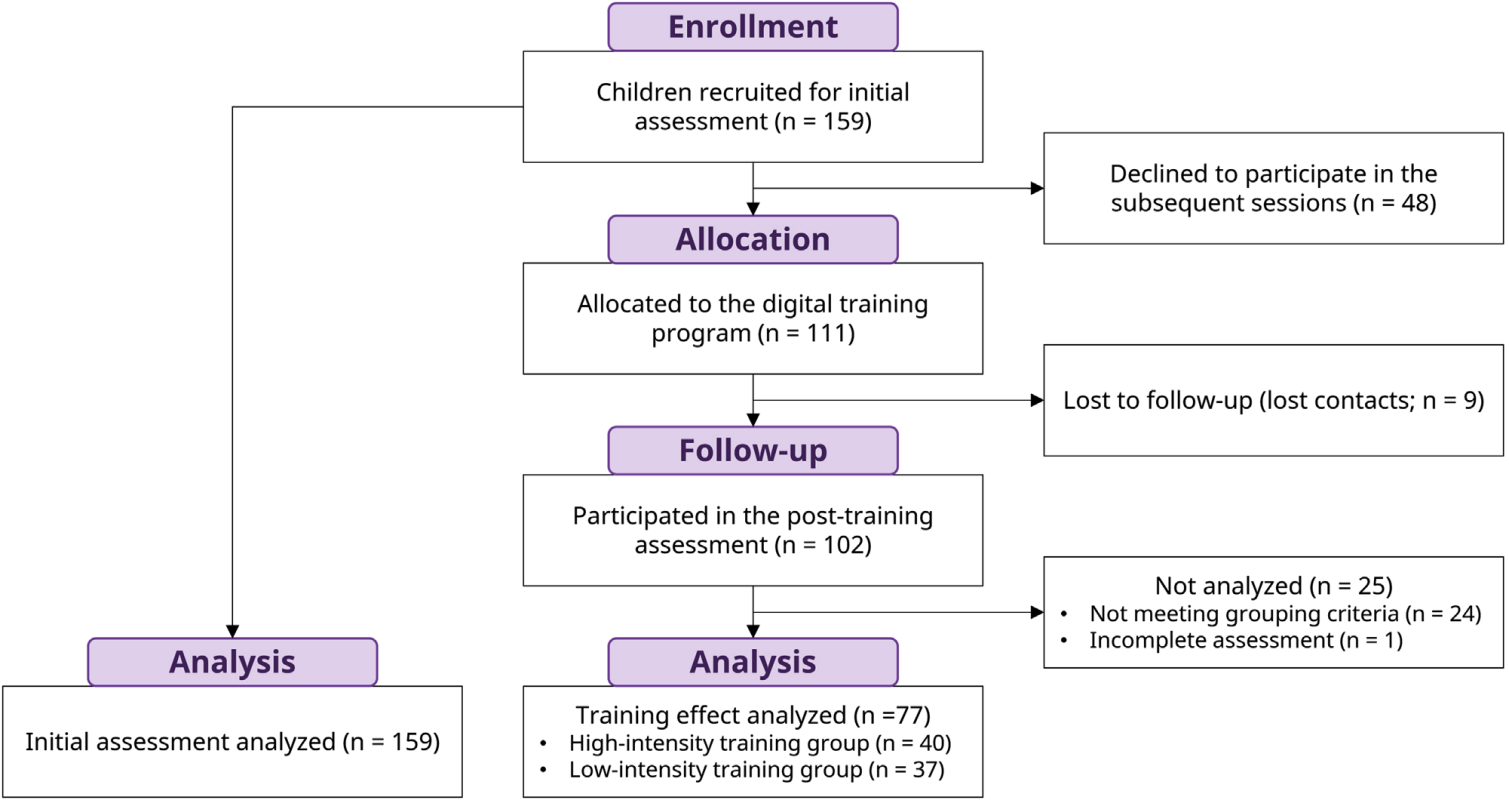
Flow chart of the process of participants through the study

The study included a total of 159 first and second graders (82 females and 77 males), with an age range from 6.41 to 8.71 (*M* = 7.66, *SD* = 0.47). In Taiwan, formal math curriculums are not introduced until the elementary school stage. Thus, our participants were in the starting stage of mathematical learning. To avoid data being nested and to include children from families with a wide range of socioeconomic status, we recruited participants individually from multiple school districts in Taipei, Taiwan using mailing to schools and posting at community groups rather than examining all students within one classroom. The sample size in this study was beyond the desired number of 148, as derived from a prospective power‐calculation using the software G*Power to reach the power of 1 − *β* = .95 at *α* = .05 based on the effect size (*r* = −.29) found in the study of Gunderson et al. (2017) who recruited participants from a similar age range with our current study. All the study protocols were approved by National Chengchi University Review Board. All participants were volunteers and were treated under the guidelines of the declaration of Helsinki. Before participating in the experiment, informed written consent was obtained from the legal guardian of each participant. After completion of each session of initial and post‐assessment, participants received monetary compensation for the completed session. The flow of participants’ progression is shown in Figure 1.

### 2.2 Overall procedure

**FIGURE 2:**
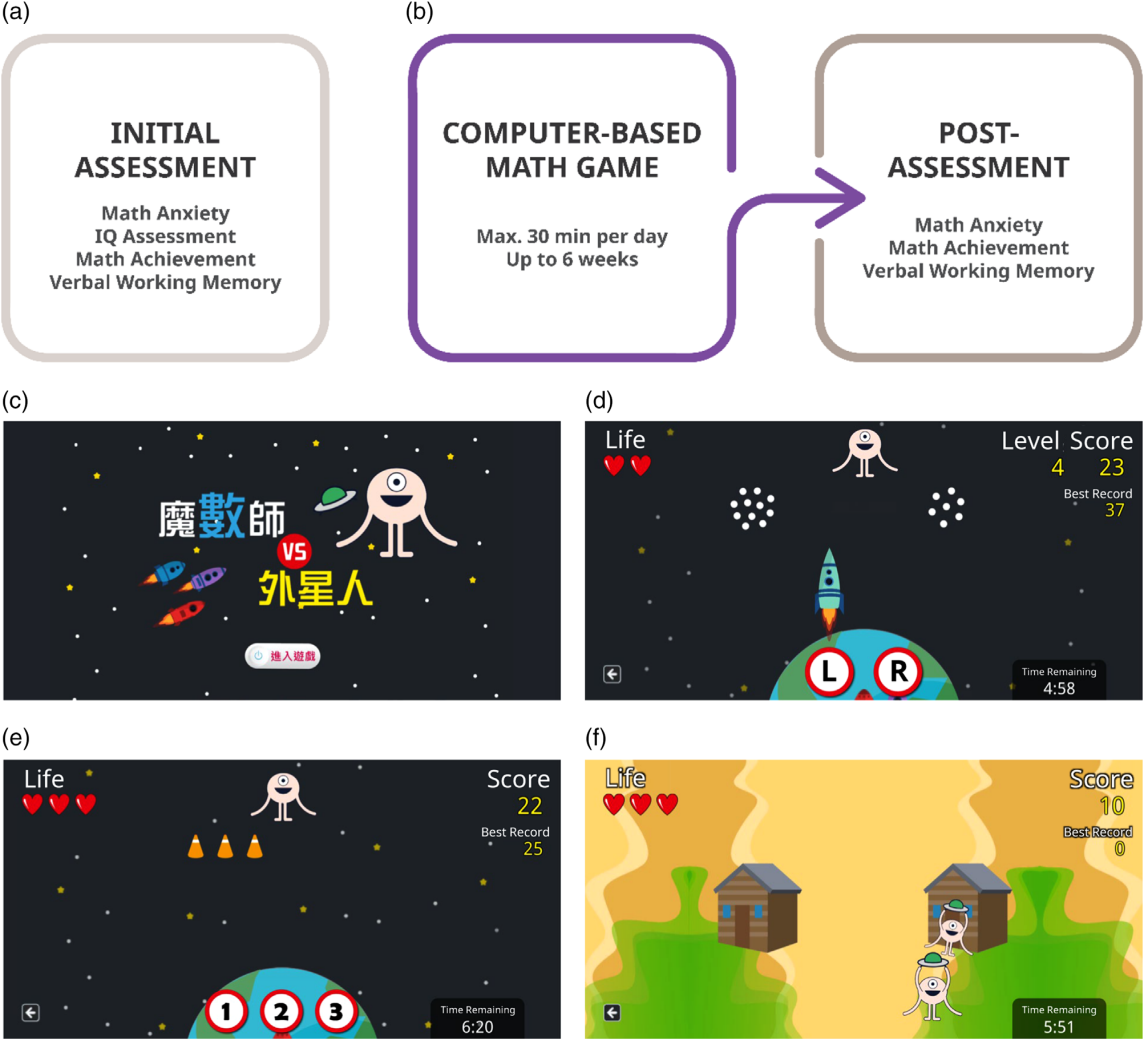
Overall study design. (a) All children underwent math anxiety and cognitive assessments. (b) After completing the initial assessment, volunteered children underwent math game training and post‐assessment for math anxiety and cognitive measurements for subsequent analysis. (C) A screenshot of the login screen of the training program. (d–f) Modules 1 to 3 of the math training game, respectively. While the actual program was presented in Traditional Chinese, the English‐translated version was provided for illustration purposes

Figure 2a,b depicts the overall procedure. All the participants underwent the initial assessment in which demographic and cognitive measures were obtained. In addition to the math anxiety level, the cognitive measures of intelligence quotient (IQ), mathematics performance, and working memory capacity were obtained. Because randomization is not preferred by volunteered parents and children, the current study was conducted with a quasi‐experiment design due to ethical concerns. After the initial assessment, 111 children volunteered to participate in the subsequent training session in which they were instructed to play a computer‐based mathematical training game. To raise ecological validity for real‐world educational application, children who participated in the training session were encouraged to play the game at home in a self‐initiated and self‐aided manner for 6 weeks. Children who had participated in the training session were further categorized as high‐ and low‐intensity training groups based on the time they were exposed to the game (the grouping criteria see Section 3.3). After the training, math anxiety, math achievement, and working memory were administered again in the post‐assessment session. Due to some children being lost to follow‐up, failing to meet the grouping criteria, or failing to complete the assessments, a final sample of 77 children, consisting of 40 children in the high‐intensity group and 37 children in the low‐intensity group with their time interval between pre‐ and post‐tests manually matched, were analyzed for the training effects (Figure 1). The sample size was beyond the number of 24, as derived from prospective power calculation based on the effect size of $\eta_{p}^{2}$ = 0.14 derived from the math anxiety remediation study of Supekar et al. (2015) to reach the power of 1 − *β* = .95 at *α* = .05.

### 2.3 Math anxiety assessment

To assess children's math anxiety levels, we adapted Child Math Anxiety Questionnaire (CMAQ) (Ramirez et al., 2013), which is an eight‐item battery assessing first and second graders’ math anxiety levels. This test was revised from Mathematics Anxiety Rating Scale for Elementary children (Suinn et al., 1988) with age‐appropriate math problems. In the CMAQ, five items asked about children's attitudes toward solving arithmetic problems (e.g., “There are 13 ducks in the water, there are 6 ducks on land, how many ducks are there in all?”), whereas the remaining three items asked about their feelings when confronting math at school (e.g., “being called on by a teacher to solve a math problem on the board”). Participants answered each question using a scale from 1 to 5 that featured a *calm face* on the number of 1 and a *very nervous face* on the number of 5. For each participant, the sum of the answers to the eight questions was computed as a math anxiety index. This questionnaire took about 1 min to complete. To provide internal reliability of the math anxiety scale, we computed Cronbach's alpha on the initial assessment of the total sample of 159 children. The alpha coefficient was .77 (95% CI [.72, .82]), indicating high internal consistency of the math anxiety measurement. Cronbach's alpha administered on the 77 children being analyzed for training effects was .75 (95% CI [67, .83]) at the initial assessment and .83 (95% CI [.77, .88]) at the post‐training assessment. These assessments showed that the internal consistency was stable over time.

### 2.4 Cognitive assessments

#### 2.4.1 IQ Assessment

IQ was assessed using the Chinese version of the Wechsler Intelligence Scale for Children (WISC‐IV), which can be administered to participants aged between 6 and 16 (Wechsler, 2004). Because (i) variability across subtests attenuate samples when split by Full‐Scale IQ (Detterman & Daniel, 1989), (ii) *Block design* and *Vocabulary* were two subtests that showed the highest correlations with Full‐Scale IQ (Facon, 2006), and (iii) the time constraints and efforts on young children participants, only the *Vocabulary* and *Block Design* subtests were administered as the IQ assessments, which took roughly 15 min. The scaled scores of the two subsets were applied as IQ measures.

#### 2.4.2 Mathematical achievement

To assess the more complex high‐order numerical knowledge that requires conceptual knowledge of mathematical rules and principles (Berch, 2005), we conducted the Basic Mathematical Core Skills Tests (BMCST) (Hung & Lian, 2015), a comprehensive paper‐and‐pencil assessment. This test is often used in Taiwan to estimate school‐age children's mathematical achievement and for the diagnosis of developmental dyscalculia. The entire battery consisted of three timed subsets assessing numerical knowledge, simple arithmetic, and complex arithmetic. The numerical knowledge subtest, measuring school‐taught numerical knowledge at the conceptual level, comprised four tasks. The first task was number counting, which contained seven multiple choice questions (e.g., “when starting counting from 10, which of the numbers, 32 and 59, will come first?”), with a time limit of 2 min. The second task was number identification with 11 questions. For the first eight items, participants were orally given a multi‐digit number and required to write down or select the corresponding number symbols. For the remaining three items, participants were presented with verbal number words and required to write down corresponding Arabic digits. A 10‐s limit was set for the number word items. The third task was numerical ordering which contained five items requiring number series completion (participants had to fill in the missing part in a sequenced number string, for example, “3, _, 9, 12”) within 1 min. The last task was to compare digits of different place values (e.g., “choosing which 4 is greater in 3427 vs. 1845”) composed of six problems to be answered in 1 min. The simple arithmetic battery from the BMCST subtest comprised single‐ and two‐digit addition and subtraction tasks, with 24 problems for single‐digit operations to be answered in 2 min and eight problems for two‐digit operations to be answered in another 2 min. For the two‐digit operations, no carrying or regrouping was required, and the sum would not exceed 99. Participants were instructed to solve the problems as quickly and accurately as possible. The complex arithmetic battery comprised seven 2‐digit addition and seven 2‐digit subtraction that required carrying and regrouping strategies, and eight 3‐digit plus/minus 2‐digit problems. Each subtest of the BMCST had provided high test reliability with Cronbach's alpha values of .87 to .90, as calculated based on the Taiwanese Grade 2 sample (Hung & Lian, 2015). Because this assessment only provided national norms for Grade 2 to Grade 6, the number of correctly answered problems rather than the standardized score from the numerical knowledge subtest was computed as an index of children's numerical knowledge, and the number of correctly answered problems of both simple and complex arithmetic batteries (hereafter “arithmetic calculation subtest”) as an index of their calculation skill.

#### 2.4.3 Verbal working memory

To assess participants’ working memory capacity, we used the *Digit Span* subset score, which is the composite of the forward and backward span tests on the Chinese version of WISC‐IV (Wechsler, 2004). Specifically, the forward digit span measured immediate verbal short‐term memory, and the backward digit span task measured executive attention (Engle, 2002). The scaled score of this subset was used as a working memory measure. The entire test took about 10 min.

### 2.5 Game design and the designing principles

A digital game‐based training program named “Igo Invasion” (Figure 2) was developed for remediation of math anxiety and implemented to be installed on any PC or Android‐system tablet. The training program was designed as a game as it (1) used character settings and colorful presentation to increase attractiveness; (2) included multiple elements for boosting motivation; and (3) implemented adaptive and personalized design based on learner's performance (Plass et al., 2015). During the training session, participants were encouraged to play the game for a maximum of 30 min a day (10 min for each module) at home. The time limit set per day was to avoid players being obsessed with the game. A timer that controlled the daily playing time was always visible to the players during the game and trackable by the experimenters. The hypothetical principles pertinent to the remediation of math anxiety are illustrated below.

1. Enhancing Math Learning
  The first principle was to enhance math learning to help participants repeatedly and intensively expose to math problems. Based on previous literature that showed best training effects (Nemmi et al., 2016; Obersteiner et al., 2013), we selected the approach to include multiple core numerical skills into the training. The game was composed of three modules. The first module was designed to enhance numerical quantity representation (Figure 2d). In this module, participants practiced judgment over numerical quantities, with adaptation implemented using increasing difficulty by decreasing the numerical distance of two compared quantities. In the second module, players practiced performing simple arithmetic tasks and the association between quantities and symbols (Figure 2e). Problem difficulty in this module adapted by increasing problem size. In the final module, participants practiced incremental counting and working memory (Figure 2f). The adaptive dimension of this module was the loading of working memory capacity.
2. Automatizing Math Performance
  Because math anxiety could interact with speed pressure and negatively impact on performance (Caviola et al., 2017), we aimed to enhance mathematics fluency as another designing principle in order to interfere with the link between math anxiety and speed pressure. To achieve the goal, for each module, as the players improved, the game difficulty would increase by speeding up the problem presentation.
3. Maximizing Motivation
  The essential designing principle in exposure‐based training is to maximize children's learning motivation and attention. To provide sufficient positive reinforcement in the educational game, we adopted role‐playing and adventure game‐like genres (Kalmpourtzis, 2018). In the game, an alien Igo would attack the earth with different quantities of materials. Participants, playing the character of “Super Math‐Magician,” had to stop the attack by choosing the correct answer. As shown in Figure 2d, in Module 1, the materials were two clusters of stars with different quantities. The players were instructed to choose the cluster with a greater number before the stars landed on the earth. In Module 2 (Figure 2e), the materials were weapons, and the players had to choose the corresponding digit of how many weapons were still needed to make a 5, 10, or 15 by performing simple subtraction. In Module 3 (Figure 2f), multiple Igos with flying saucers would first enter one of the two houses on the screen one at a time, followed by more Igos entering the other house. Afterward, some Igos would move from one house to the other. Players had to count and maintain how many Igos were in each house and choose the one with the greater number. This task not only required simple addition and subtraction skills of the players but also working memory to maintain numbers. For all the three modules, players would receive positive feedback once their response to the problem was correct. Players also received different numbers of stars as medals for solving the problems depending on the difficulty.

Before the training session started, a printed manual of how to install the game on tabloid and PC was provided to the parent of each participating child. To make sure each child understood how to play the game, a trained research assistant gave instructions before the training session. During the training session, the research assistant would monitor the game playing time of the children through the online logging system. Once a child lagged, the research assistant would contact the parent to encourage the child to maintain compliance.

## 3 RESULTS

### 3.1 Normative results

The descriptive demographic data and cognitive assessments of the children comprising age, math anxiety, scaled IQ measures, math skills (both arithmetic calculation and numerical knowledge), and verbal working memory are presented in Table 1. Because the children were assessed at different time points, we further separated children into End of Grade 1, Middle of Grade 2, and End of Grade 2, and examined group differences of each measurement. A Chi‐square test indicated that there was no difference in sex ratio among the three groups ($\chi^{2}$(2) = 3.18, *p* = .204). There were significant group differences in math anxiety (*F*(2,156) = 5.45, *p* = .005, $\eta_{p}^{2}$ = .07), arithmetic calculation (*F*(2,156) = 22.99, *p* < .001, $\eta_{p}^{2}$ = .23), and a marginal effect in numerical knowledge (*F*(2,156) = 2.98, *p* = .054, $\eta_{p}^{2}$ = .04). Post hoc comparisons using Tukey's HSD suggested that group differences in math anxiety were primarily elicited from children of the middle of Grade 2 showing a higher level of math anxiety than children assessed at the end of Grade 2 (*p* = .007). Group differences in calculation were primarily elicited from the enhancement between children from Grade 1 and those from the middle of Grade 2 (*p* < .001). No other group differences were observed. Because of the tight age range and the limited sample size, participants from the three groups were pooled for subsequent analyses.

**TABLE 1:** Descriptive characteristics and initial assessment performances of the participating children

Variable	Overall	End of Grade 1 (G1)	Middle of Grade 2 (G2a)	End of Grade 2 (G2b)	*p* ^a^	Post hoc comparisons^b^
*N* (female/male)	82/77	17/19	44/47	21/11	.204	–
Age	7.66 (0.47)	7.15 (0.38)	7.72 (0.35)	8.05 (0.40)	<.001	G1 < G2a < G2b
Math anxiety	18.18 (6.83)	17.06 (6.54)	19.60 (6.50)	15.38 (7.18)	.005	G1 = G2a > G2b
Block design	12.30 (2.85)	12.08 (2.49)	12.58 (2.85)	11.75 (3.18)	.319	G1 = G2a = G2b
Vocabulary	11.69 (2.78)	12.56 (2.96)	11.47 (2.79)	11.34 (2.42)	.103	G1 = G2a = G2b
Verbal working memory	11.15 (2.49)	11.22 (2.62)	11.38 (2.34)	10.41 (2.69)	.158	G1 = G2a = G2b
Numerical knowledge	21.11 (5.45)	19.31 (6.15)	21.38 (4.72)	22.34 (6.21)	.054	G1 = G2a = G2b
Arithmetic calculation	40.71 (10.75)	31.47 (11.19)	42.69 (8.68)	45.47 (9.74)	<.001	G1 < G2a = G2b

*Note*: Mean and standard deviation (in parentheses) values are reported.

^a^ *p* values of the chi‐squared test (sex‐ratio difference) or one‐way ANOVA (age and the remaining assessments) examining differences among the three groups of children.

^b^ Post hoc multiple comparisons based on the Tukey's HSD tests, with a significance level set at *α* = .05.

### 3.2 Math anxiety and its relation to math achievement

**FIGURE 3:**
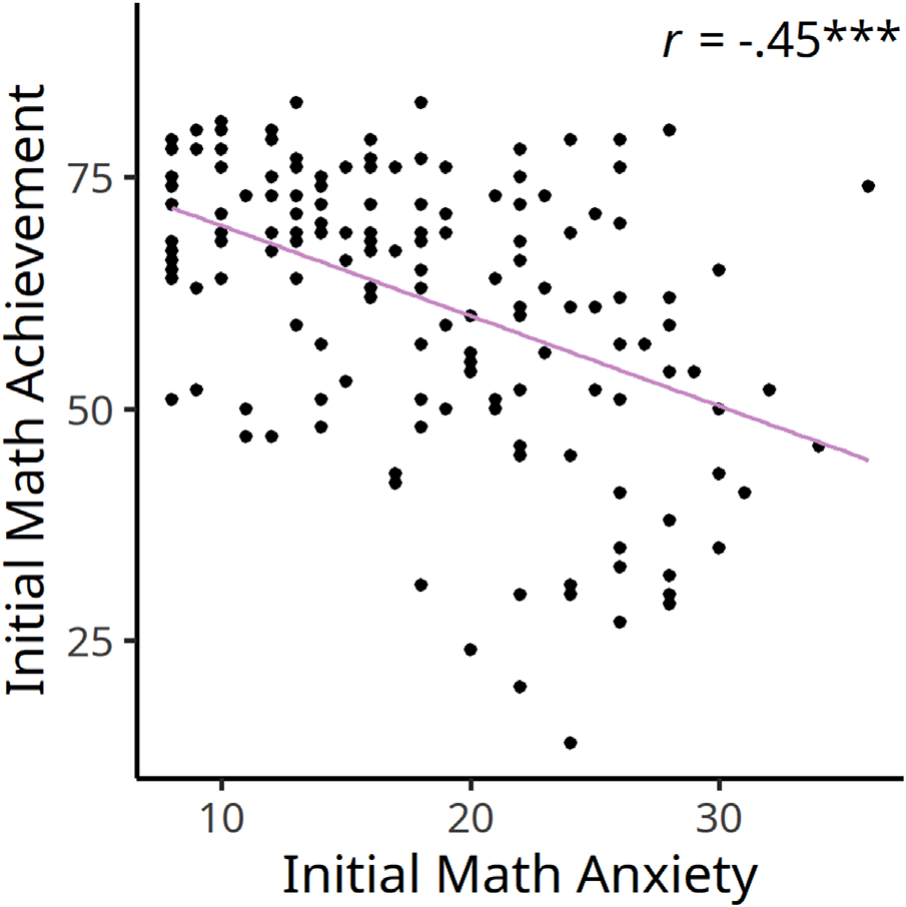
Math anxiety was negatively correlated with math achievement at the initial assessment. ***p < .001

We examined the relationship between math anxiety and math achievement. Zero‐order correlation between the initial math anxiety and math achievement was conducted and is presented in Figure 3. Results revealed that initial math anxiety was negatively correlated with the initial performance in the BMCST (*r*(157) = −.45, *p* < .001, 95% CI [−.56, −.31]). After controlling for age and the IQ measures using hierarchical regression analysis, the relation between math anxiety and math achievement remained significant (standardized *β* = −0.29, *t* = −4.66, *p* < .001, ∆*R* ^2^ = .08). We further examined the relations in each of the math achievement subtests. Negative relations with math anxiety were observed in both numerical knowledge (*r*(157) = −.43, *p* < .001, 95% CI [−.55, −.43]) and arithmetic calculation (*r*(157) = −.40, *p* < .001, 95% CI [−.52, −.26]) subtests. While controlling for age and the IQ measures, the negative correlations remained significant (numerical knowledge: standardized *β* = −0.28, *t* = −4.19, *p* < .001, ∆*R* ^2^ = .07; arithmetic calculation: standardized *β* = −0.26, *t* = −3.97, *p* < .001, ∆*R* ^2^ = .06).

### 3.3 Digital math intervention reduced math anxiety in first and second graders

To raise ecological validity, children who participated in the training session were encouraged to play the training game at home after school 30 min a day, at least 3 days a week for 4 to 6 weeks. Children who maintained compliance and participated in the full training session for more than 6 h in total were included as the high‐intensity training group. Children who were exposed to the game for less than 2 h with a matched interval between the initial and post‐training assessment were included as the low‐intensity training group. These criteria resulted in a sample of 40 children in the high‐intensity group and 37 children in the low‐intensity group. Demographic data of the two groups were examined and are presented in Table 2. Because the two groups were generated based on self‐selection in a quasi‐experiment design, we first investigate whether the two groups differed in cognitive assessments before training. Since the sample distribution of some variables did not fulfill the normality assumption, we conducted nonparametric Kruskal–Wallis tests for each cognitive measurement. None of the math anxiety, math achievement, working memory, or IQ measures was differed between the two groups at the initial assessment (Table 2), suggesting that the two groups should be highly homogeneous in all the baseline characteristics. To further ensure the role‐playing and adventure game‐like settings in the training program did not favor either male or female participants, we examined sex differences in training duration. Results suggested no difference between male and female participants in any of the training groups (high‐intensity: Kruskal–Wallis $\chi^{2}$(1) = 1.88, *p* = .170; low‐intensity: Kruskal–Wallis $\chi^{2}$(1) = 2.06, *p* = .151).

**TABLE 2:** Descriptive characteristics and initial assessment performances of the low‐ and high‐intensity training groups

Variable	Low intensity	High intensity	$\chi^{2}$	*p*
*N* (female/male)	24/13	27/13	0.00	.998
Age	7.77 (0.43)	7.64 (0.51)	0.45	.501
Math anxiety	17.97 (6.57)	16.25 (5.98)	1.32	.250
Block design	11.86 (2.83)	12.57 (2.85)	1.08	.298
Vocabulary	11.14 (2.61)	11.85 (3.07)	2.04	.153
Verbal working memory	11.38 (2.52)	11.20 (2.89)	0.11	.735
Numerical knowledge	21.30 (5.23)	22.32 (4.55)	0.81	.368
Arithmetic calculation	43.38 (9.61)	41.67 (8.62)	1.53	.217
Training duration (h)	0.35 (0.59)	10.35 (2.90)	58.70	<.001
Between‐assessment interval (days)	49.46 (18.48)	55.70 (15.14)	3.40	.065

*Note*: Mean and standard deviation (in parentheses) values are reported.

**FIGURE 4:**
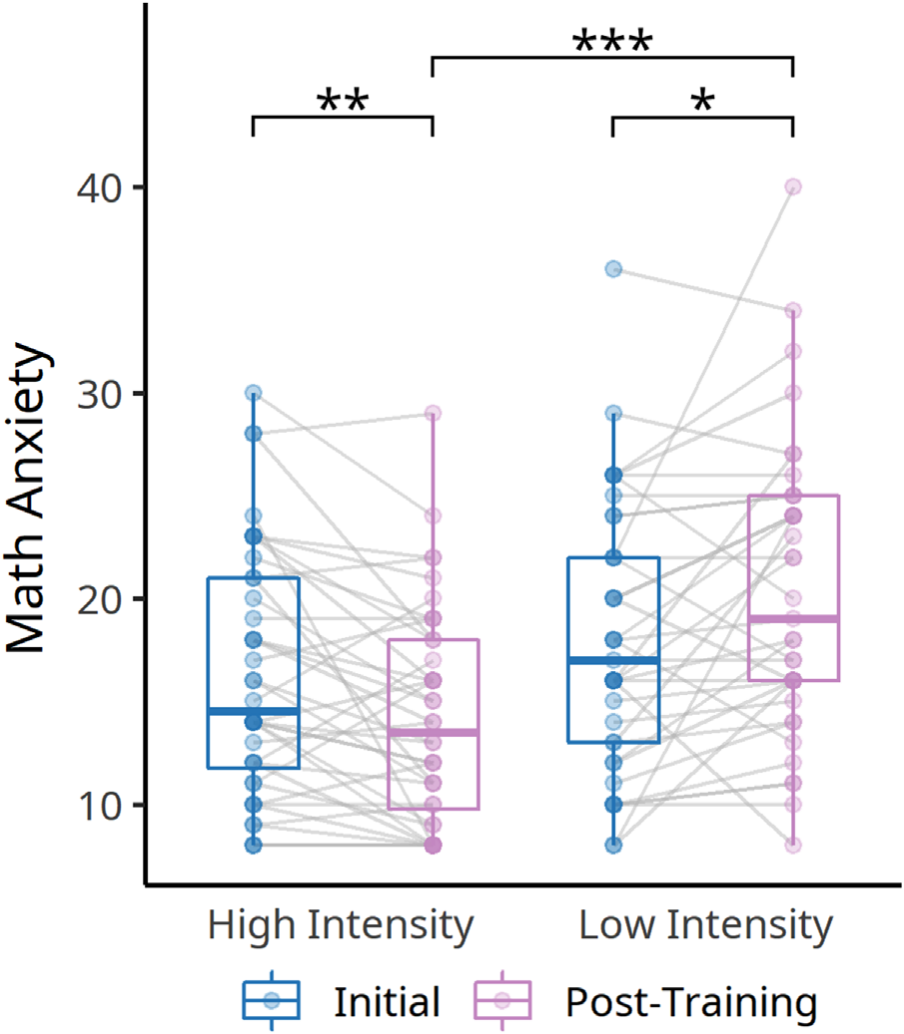
Training effects on math anxiety within the high‐ and low‐intensity groups. There was a significant interaction between time (initial vs. post‐training) and training intensity. The high‐intensity training group showed a reduced math‐anxiety level after training, whereas the low‐intensity group showed an opposite pattern. ***p < .001, **p < .01, *p < .05

Next, we examined the remediation effect in math anxiety elicited by the computer‐based training program. Since the normality assumption was not met in the data, we conducted nonparametric analyses of the factorial design using ANOVA‐type statistics (ATS) (Noguchi et al., 2012) on math anxiety, with time (initial vs. post‐assessment) as the within‐participant factor and training intensity (low vs. high) as the between‐participant factor implemented in R package *nparLD*. Two‐way interaction between time and training intensity was detected (ATS *F* = 14.11, *df* = 1, *p* < .001; Table 3, Figure 4). Simple main effect analyses indicated that the interaction was driven by math anxiety showing a significant reduction in the high‐intensity group after the training (ATS *F* = 8.49, *df* = 1, *p* = .004), whereas math anxiety slightly increased in the low‐intensity group (ATS *F* = 6.38, *df* = 1, *p* = .012). After training, the high‐intensity group showed lower math anxiety than the low‐intensity group (Kruskal–Wallis $\chi^{2}$(1) = 14.80, *p* < .001). Considering the variation of individuals at the initial assessment, we conducted an ANCOVA to compare post‐assessment math anxiety between the two groups adjusted by both initial math anxiety and math performance. Results indicated the training‐related math anxiety relief persisted after controlling for mathematical anxiety and performance at baseline (*F*(1,73) = 22.20, *p* < .001, $\eta_{p}^{2}$ = .23).

**TABLE 3:** Results of nonparametric analysis on the remediation effect in math anxiety

Effect	*df*	F */* $\chi^{2}$	*p*
**Effects of training intensity and time**			
Overall participants			
Group	1	8.51	.004
Time	1	0.01	.910
Group × time	1	14.11	<.001
Group = high intensity			
Time	1	8.49	.004
Group = low intensity			
Time	1	6.36	.012
Time = post‐training			
Group	1	14.80	<.001
**Effects of initial math anxiety and time**			
High‐intensity training group			
Initial math anxiety	1	80.67	<.001
Time	1	9.05	.003
Initial math anxiety × time	1	3.57	.059
Initial math anxiety = high			
Time	1	10.31	.001
Initial math anxiety = low			
Time	1	1.79	.181
Time = post‐training			
Initial math anxiety	1	16.30	<.001

We further examined whether different types of math anxiety (problem solving‐related and school setting‐related math anxiety) showed differential remediation effects by conducting a parallel analysis on each math anxiety subscale. The results revealed a significant two‐way interaction effect on the problem‐solving related math anxiety subscale (ATS *F* = 11.54, *df* = 1, *p* < .001) and a marginally significant interaction on the school setting‐related math anxiety subscale (ATS *F* = 3.84, *df* = 1, *p* = .050). To examine whether sex bias existed in the training‐associated relief of math anxiety, a three‐way ANOVA‐type nonparametric statistic for training intensity, time, and sex was conducted. Results indicated the math anxiety relief did not differ between male and female participants (ATS *F* = 0.05, *df* = 1, *p* = .817).

### 3.4 Digital intervention reduced math anxiety only in children with high math anxiety

**FIGURE 5:**
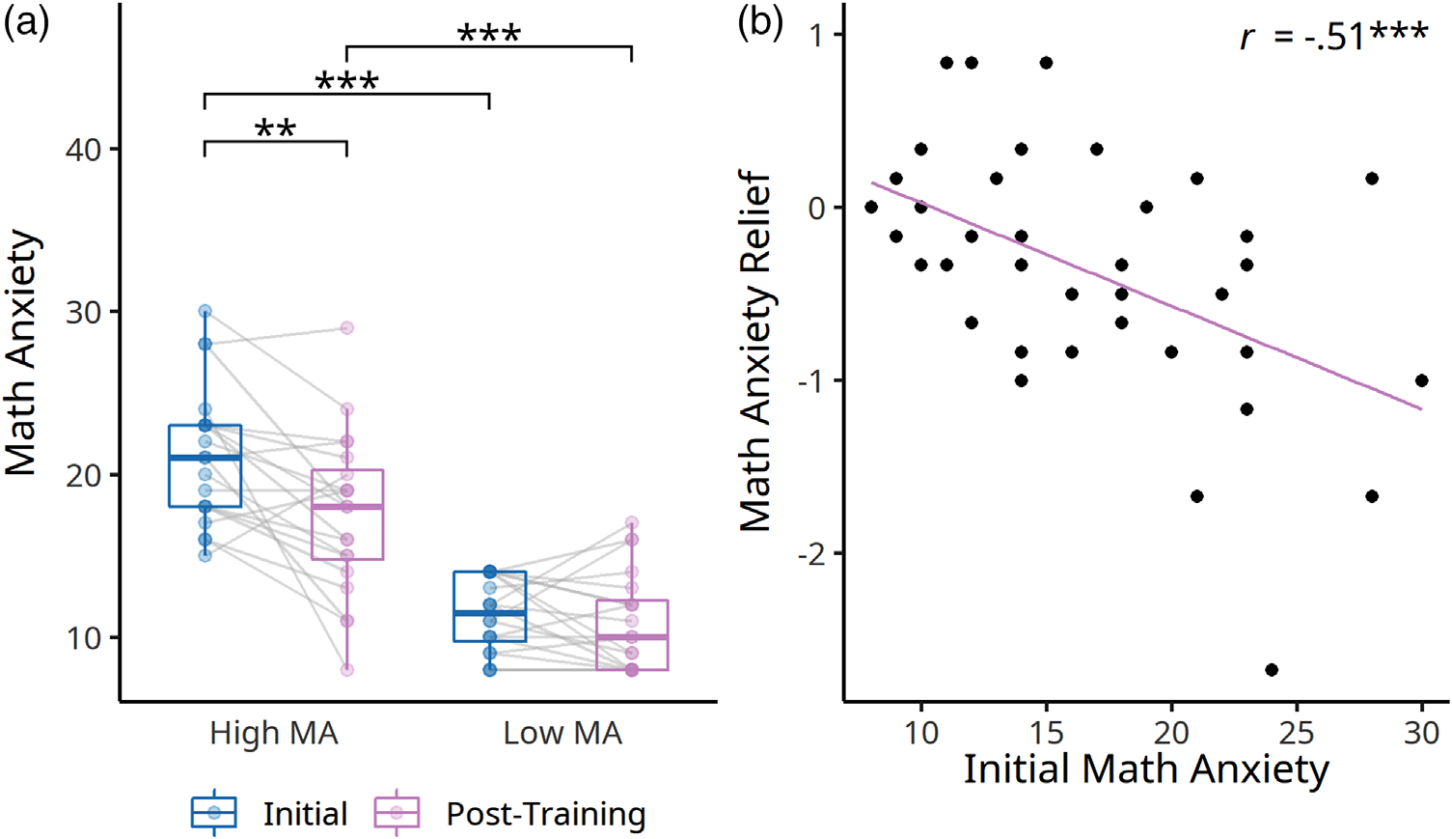
Relations between initial math anxiety (MA) and training‐related math anxiety changes in the high‐intensity training group. (a) There was a marginal interactive effect on math anxiety between time (initial vs. post‐training) and group (high vs. low MA at baseline), with only the high‐anxiety group showing a reduction in math anxiety after training. (b) There was a negative correlation between initial math anxiety and training‐related math anxiety relief. ***p < .001, **p < .01

To further investigate whether children who suffered from different levels of math anxiety showed differential math anxiety remediation effects, we divided children into high and low math anxiety groups based on the initial math anxiety of the high‐intensity group using a median split. This procedure resulted in 20 high and 20 low math anxiety children in the high‐intensity training group. Results revealed a marginally significant interaction between the initial math anxiety level (high vs. low) and time (ATS *F* = 3.57, *df* = 1, *p* = .059; Table 3, Figure 5a). Simple effect analyses indicated that training‐associated math anxiety remediation was detected only in the high math anxiety group (ATS *F* = 10.31, *df* = 1, *p* = .001) but not the low anxiety group (ATS *F* = 1.79, *df* = 1, *p* = .181). Note that the training durations of the high and low math anxiety groups were not different (Kruskal–Wallis $\chi^{2}$(1) = 2.46, *p* = .117), suggesting that the math anxiety alleviation did not result from their unequal engagement in training. To ensure such an interaction was not due to the regression‐to‐the‐mean effect, we performed the same analyses on the low‐intensity training group. The median‐split approach resulted in 19 high and 18 low math anxiety participants. Importantly, the group‐by‐time interaction pattern of the low‐intensity training group was different from the high‐intensity training group (Figure S1A, Table S1) than those children with high math anxiety remained highly math‐anxious (ATS *F* = 0.51, *df* = 1, *p* = .474), whereas those with low math anxiety showed increased math anxiety (ATS *F* = 7.64, *df* = 1, *p* = .006), implying the math anxiety remediation was contributed by the digital training rather than confounded by the regression‐to‐the‐mean.

We then examined whether there was an individual difference in math anxiety relief. Math anxiety relief was calculated by first subtracting initial math anxiety from post‐training math anxiety and then divided the given value by the standard deviation of initial math anxiety. We assessed the correlation between math anxiety relief and initial math anxiety for children from the high‐intensity training group (Figure 5b). Critically, children with higher initial math anxiety tended to experience a greater reduction in math anxiety after active engagement toward the digital math training program (*r*(38) = −.51, *p* < .001, 95% CI [−.71, −.24]). A parallel analysis was conducted on children from the low‐intensity training group to confirm the specificity of such a relation. Results indicated the initial math anxiety was not significantly associated with the changes of math anxiety in the children who minimally attended the training, if at all (*r*(35) = −.30, *p* = .070, 95% CI [−.57, .03]; Figure S1B).

### 3.5 Digital math intervention improved working memory capacity and math performance in first and second graders

We then examined whether verbal working memory capacity was improved by the 6‐week digital training on working memory performance. The interaction between group and time was marginally significant (ATS *F* = 3.47, *df* = 1, *p* = .063; Table 4), with the high‐intensity training group showed increased working memory capacity after training (ATS *F* = 4.83, *df* = 1, *p* = .028), whereas the low‐intensity group did not show the effect (ATS *F* = 0.11, *df* = 1, *p* = .736). Yet, the post‐training working memory between the high‐ and low‐intensity training groups was not statistically different (Kruskal–Wallis $\chi^{2}$(1) = 1.48, *p* = .223).

**TABLE 4:** Results of nonparametric analysis on the improvement in working memory capacity

Effect	*df*	F */* $\chi^{2}$	*p*
Overall participants			
Group	1	0.28	.595
Time	1	2.29	.130
Group × time	1	3.47	.063
Group = high intensity			
Time	1	4.83	.028
Group = low intensity			
Time	1	0.11	.736
Time = post‐training			
Group	1	1.48	.223

Parallel analyses were conducted on children's math achievement. Similarly, the interaction effect between group and time was significant (ATS *F* = 9.23, *df* = 1, *p* = .002; Table 5). Results of simple effects suggested that the high‐intensity group exhibited increased math achievement after training (ATS *F* = 16.54, *df* = 1, *p* < .001), whereas the low‐intensity group showed no difference in math achievement between the initial and post‐training assessment (ATS *F* = 0.02, *df* = 1, *p* = .888). The post‐training difference in overall math achievement between these two groups, however, did not reach significance (Kruskal–Wallis $\chi^{2}$(1) = 1.75, *p* = .186). We further examined whether the two math achievement subtests (numerical knowledge and arithmetic calculation) showed differential training effects. The results revealed that for the numerical knowledge subtest, there was no training intensity‐by‐time interaction (ATS *F* = 0.63, *df* = 1, *p* = .428; Table 5). The arithmetic calculation subtest, in contrast, did show the interaction profile (ATS *F* = 9.01, *df* = 1, *p* = .003; Table 5) such that only the high‐intensity group showed improvement in calculation after training (ATS *F* = 13.81, *df* = 1, *p* < .001) but not the low‐intensity group (ATS *F* = 0.01, *df* = 1, *p* = .931). No difference was detected between these two groups at post‐assessment in either the numerical knowledge subtest (Kruskal–Wallis $\chi^{2}$(1) = 2.30, *p* = .130) or the calculation subtest (Kruskal–Wallis $\chi^{2}$(1) = 0.58, *p* = .447).

**TABLE 5:** Results of nonparametric analysis on the improvement in math achievement

Effect	*df*	F */* $\chi^{2}$	*p*
**Total scores of the BMCST**			
Overall participants			
Group	1	0.22	.641
Time	1	9.36	.002
Group × time	1	9.23	.002
Group = high intensity			
Time	1	16.54	<.001
Group = low intensity			
Time	1	0.02	.888
Time = post‐training			
Group	1	1.75	.186
**Numerical knowledge subtest**			
Overall participants			
Group	1	1.55	.214
Time	1	3.65	.056
Group × time	1	0.63	.428
Group = high intensity			
Time	1	3.24	.072
Group = low intensity			
Time	1	0.83	.361
Time = post‐training			
Group	1	2.30	.130
**Arithmetic calculation subtest**			
Overall participants			
Group	1	0.08	.774
Time	1	4.71	.030
Group × time	1	9.01	.003
Group = high intensity			
Time	1	13.81	<.001
Group = low intensity			
Time	1	0.01	.931
Time = post‐training			
Group	1	0.58	.447

BMCST, Basic Mathematical Core Skills Tests.

Finally, we examined whether individual differences of the performance gain were associated with the math anxiety profiles. Results indicated the training‐associated improvement in working memory, numerical knowledge, and arithmetic calculation was independent of the initial math anxiety level (working memory: *r*(38) = .26, *p* = .103, 95% CI [−.05, .53]; numerical knowledge: *r*(38) = .03, *p* = .840, 95% CI [−.28, .34]; arithmetic calculation: *r*(38) = .02, *p* = .909, 95% CI [−.29, .33]). Further, we did not find significant correlation between the training‐associated improvement in math achievement and the math anxiety relief (numerical knowledge: *r*(38) = −.28, *p* = .082, 95% CI [−.54, .04]; arithmetic calculation: *r*(38) = −.16, *p* = .320, 95% CI [−.45, .16]). The association between working memory improvement and math anxiety relief was not significant either (*r*(37) = −.20, *p* = .220, 95% CI [−.49, .12]) after excluding one outlier (beyond 2.5 standard deviations from the means for both measures). These results suggested that the improvement of cognitive abilities could neither predict the initial math anxiety nor the training‐associated math anxiety relief.

## 4 DISCUSSION

The current study has investigated the relationship between math anxiety and mathematical learning in children as young as first and second graders in Taiwan, a culture where students exhibit notoriously high‐level math anxiety despite their decent performance in mathematics (Mullis et al., 2012, 2016; OECD, 2013). We first demonstrated that math anxiety was negatively correlated with school‐learned mathematical attainments. To ameliorate math anxiety, we adopted a digital version of exposure‐based therapy. Importantly, our study is the first to design math anxiety intervention platform based on the core cognitive theory. Specifically, our math anxiety training program targeted core numerical skills, such as accelerating enumeration, enhancing numerical fluency, speeded calculation, counting, as well as enhancing working memory by maintaining numbers, in a motivating learning environment. Results revealed that children who underwent 6 weeks of intensive exposure to our composite digital math training showed remarkably reduced math anxiety, whereas children without or with barely limited training did not show this tendency. Critically, math anxiety remediation was particularly salient in children who suffered from high‐level math anxiety. These findings have collectively demonstrated that math anxiety has crucial effects on learning. Auspiciously, this problem can be effectively alleviated through exposure‐based digital learning therapy.

### 4.1 Negative association between math anxiety and math achievements in the East Asian culture

One of our main objectives is to investigate the math anxiety profile of young children in Taiwan. We find that children as young as first and second graders demonstrate self‐reported math anxiety. The math‐related negative emotion has already been associated with their math achievement such that children with higher level math anxiety show worse performance on mathematical assessments. These findings are inconsistent with the previous findings on German children of the same age (Krinzinger et al., 2009). In the study of Krinzinger et al. (2009), there was no association identified between children's math anxiety and single‐digit calculation skills. Our results, in contrast, are supported by several previous reports conducted with older children in other Western cultures (Gunderson et al., 2017; Ramirez et al., 2013; Suinn et al., 1988; Wu et al., 2012). For example, Suinn et al. (1988) demonstrated that fourth to sixth graders in the US showed a negative association between math anxiety scores and math performance assessed by SAT. Of second and third graders, Wu et al. (2012) reported that math anxiety levels were negatively linked to the calculation and mathematical reasoning skills, assessed by WIAT‐II. One possible explanation for the inconsistency is that math anxiety impacts only on demanding problems. Wu et al. (2012) reported that math anxiety had stronger negative impacts on mathematical reasoning than basic calculation skills. In another study, Ching (2017) followed a group of Chinese children from second to third grades and found that math anxiety had more pronounced effects on difficult mathematical problems. Similarly, we demonstrate that demanding numerical skills, such as multi‐digit addition and subtraction calculation as well as conceptual numerical knowledge, can be deteriorated by increased math anxiety levels, and these impacts can start as early as the first year of primary school.

Interestingly, a slight reduction of math anxiety between the middle and the end of Grade 2 is observed. Such a trend is in line with the previous literature (Gunderson et al., 2017; Maloney et al., 2015). In one study conducted with first and second graders, math anxiety was administered twice, first at the beginning of the year and the second in the end. There was a trend of a slight decrease in math anxiety identified between the two assessments (Maloney et al., 2015). In another study conducted with children of the same age, math anxiety was measured in the first three months of the school year and again in the last two months of the school year. Similarly, math anxiety was reduced between the two assessments (Gunderson et al., 2017). However, none of these two studies directly tested and discussed the effects. We speculate that as the school year proceeds, students gradually get used to the second‐grade school curriculum and thus demonstrate less worry about math learning. The limited sample size and cross‐sectional sample in the current study still warrant further investigation on this developmental progression.

### 4.2 Remediation effect of the digital game‐based training in math anxiety

Another major contribution is we demonstrate that after high‐intensity training for 6‐week, children show a salient reduction in math anxiety, whereas children with low‐intensity training do not show this tendency. How, then, do we interpret the training‐associated math anxiety reduction? We suspect that there are two possible sources to help children reduce math anxiety. First, it is the sustained exposure to numerical and working memory training which helps alleviate children's negative emotional reactions toward math learning. Exposure therapy is designed based on disrupting the expectation of an association between exposure to anxiety resources and aversive outcomes (Craske et al., 2014). The more the expectancy is violated during exposure, the greater the anxiety is inhibited. Additionally, children learning in traditional settings are vulnerable to anxiety sources from the environmental factors, such as peer pressure and unfriendly behaviors of teachers (Suárez‐Pellicioni et al., 2016). The pressure from peers, teachers, and parents appears to be even more severe in East Asian countries (Tan & Yates, 2011), possibly because students in these countries particularly worry about others’ perceptions when they fail (OECD, 2019). Herein, the application of exposure therapy with a computerized training protocol assists student‐centered learning of children and reduces math‐related worries through daily exposure to math practices with minimal, if any, unfavorable outcomes. Crucially, both problem‐solving anxiety and school situation‐related anxiety showed relief after the training, suggesting that our remediation not only frees up children's calculation anxiety but also transfers to children imagining situations about math at school.

Another possibility is that our protocol provides a child‐friendly game setting that serves as entertaining materials. Unlike the school mathematics curriculum, our self‐initiated and self‐aided game‐based digital program allows children to practice core numerical skills at home with minimal technical support. Such practice‐oriented materials can be less struggling and, in the meantime, more playful than homework assigned at school, and hence provide an encouraging and motivating learning environment while being pedagogically meaningful. Still, more work is needed to tease apart which resources contribute most significantly in alleviating children's negative emotions toward learning.

The remediation protocol of the current study is particularly useful for children who suffer from high‐level math anxiety. Only the highly math‐anxious children show significantly reduced math anxiety after the intervention. Moreover, the more severe their math anxiety is, the greater these children benefit from math anxiety remediation. These results are consistent with previous studies using different approaches of math anxiety intervention (Park et al., 2014; Supekar et al., 2015). Park et al. (2014) asked adult participants to write down their negative feelings and intrusive thoughts prior to solving arithmetic problems. They found that expressive writing also ameliorated arithmetic performance, and such an effect was more pronounced in the high math anxiety group (Park et al., 2014). In Supekar et al.’s (2015) one‐by‐one math tutoring intervention, math anxiety was alleviated only in children with high math anxiety but not in their low math anxiety peers. Together, these findings not only indicate that those with intense math anxiety can be treated but also provide a rough estimate of how much math anxiety can be improved. The deficits of children with high math anxiety are linked to excessive functional activations in the amygdala and other arithmetic‐associated neural circuits (Supekar et al., 2015; Young et al., 2012). These aberrant activations can be normalized to activity levels in parallel with the low math anxiety group after remediation (Supekar et al., 2015), implicating the biological bases of math anxiety and that it can be remediated through neural functional modulation.

### 4.3 Math anxiety relief was independent of the training‐associated cognitive improvement

As predicted, our composite game‐based training in the digital form also enhances children's math performance as well as working memory. In the study of Nemmi et al. (2016), combined training in number line and working memory enhances the arithmetic skills of the 6‐year‐old, whereas in Sanchez‐Perez et al. (2017), training at working memory plus calculation improves school math achievement of third‐to‐sixth graders. Collectively, these studies have demonstrated that learning outcomes can be effectively improved by composite training. Note that the cognitive components delivered in the program were primarily designed based on nonsymbolic processing, whereas the cognitive performance being assessed in the pre‐ and post‐sessions exclusively focused on symbolic processing. As pointed out by De Smedt et al. (2013) in a systematic comparison of symbolic and nonsymbolic studies in the existing literature, symbolic and nonsymbolic numerical skills exhibit very distinct profiles. Specifically, for numerical tasks on digits, results consistently showed weak performance correlates with low math achievement, whereas for numerical tasks on dots or shapes, results have been conflicting. Moreover, training over nonsymbolic approximate quantity manipulation does not guarantee transferring to symbolic calculation (Kim et al., 2018). We, therefore, believe that the cognitive enhancement in our study is due to transfer effect rather than merely practice effect.

The practices of fundamental skills using the digital platform are endowed with a relatively playful manner and can transfer to more integrative math ability. In addition, since math anxiety causes math avoidance and thereby impairs math learning (Choe et al., 2019; Foley et al., 2017), it was hypothesized that higher math anxiety might lead to worse learning outcomes by showing smaller performance gains in mathematical achievement. However, such correlation is not found in the current study, and children have improved at math no matter if they are bearing a heavy burden of math anxiety. This suggests that our digital game‐based learning should have provided a friendly environment for math learning, and children with and without math anxiety benefit equally from such learning.

On the other hand, we have found the training‐associated math anxiety relief is uncorrelated with the performance gains, indicating such relief is less likely caused by the improved problem‐solving skills. Rather, the finding supports that mere sustained exposure to mathematical materials could be effective against excessive math anxiety. This is in agreement with Supekar et al. (2015) that the prolonged exposure to math impacts directly on remediating math anxiety for students with high math anxiety, as students from the high and low math anxiety groups show similar performance gain in arithmetic. Yet, due to the close relations among math anxiety, math performance, and working memory (Ashcraft & Krause, 2007), we suspect that the benefits from relieving math anxiety and cognitive improvement would be multiplicative in the long run. Nevertheless, the timescale of the current study does not allow us to investigate such possibility. Future studies for examining the long‐term effects of digital game‐based training concerning both achievements and emotions are expected.

### 4.4 Limitations

While proposing an effective strategy for math anxiety remediation, we acknowledge that there are potential limitations in the current study. The most crucial limitation is this study was implemented in a quasi‐experimental design, with the experiment and comparison groups being defined based on participants’ engagement during the training session rather than random assignment due to ethical concerns. This design can lead to potential problems. First, participants’ learning motivation and interests toward math learning could be introduced as confounding variables due to the self‐initiated nature of the digital training. Although we have controlled the high‐ and low‐intensity groups to be as similar as possible and affirmed all the initial cognitive measurements not being different, it is still possible that the two groups differ in learning motivation. However, Gunderson et al. (2017) have reported learning motivation does not predict math anxiety in first and second graders in the first three months of the school year. We, therefore, suspect that differences in the motivation of participants are plausibly negligible. Another problem introduced by the quasi‐experimental design could be the regression‐to‐the‐mean effect due to the lack of a control group. Yet, we have demonstrated that math‐anxious children have shown math anxiety relief only when they are highly engaged in our digital training, whereas those with minimal engagement remained math‐anxious. These findings suggest the remediation effect on math anxiety in the current study should likely be a real effect rather than a regression artifact.

Another limitation is it would be arguably better to include a control group with a different kind of training. An active control group can help eliminate placebo effects and locate the sources of the remediation effect by elucidating whether it results from prolonged exposure to math materials or the motivating game‐based environment. Nevertheless, Szűcs and Myers (2017) argued that including target‐irrelevant training and comparing it with target‐relevant training can largely exaggerate group differences by inserting more target‐related instructions in the experimental group. Despite that the current study lacks controlled training, our findings have provided an estimate of the efficacy of math anxiety improvement. Further interventions with equivalently entertaining and motivating training at a similar difficulty level are still expected.

## 5 CONCLUSION

In conclusion, this study speaks to parents and educators to pay attention to students’ perceptions, emotions, and attitudes toward learning, even in the cultures where students are generally competent in mathematics. Negative attitudes toward learning can cause disruptions to students’ learning and further discourage students from being well performed (Rattan et al., 2012). Fortunately, those who struggle with math anxiety can be relieved from short‐term intensive intervention. Our study provides novel empirical evidence that a computer‐assisted composite training of mathematics is effective for reducing math anxiety in first and second graders with severe math anxiety. Such enhancement provides broad implications and boosts efforts to bridge the gap between research and application. In particular, our training approach potentially serves as an economically efficient and easily implemented solution for learning‐associated emotional deficits. Our study highlights the unique potential of this approach to the advancement of mathematical learning in education practice. More broadly, the cognitive framework established here is likely useful for developing targeted training and intervention programs, and it can be generalized to other forms of skill learning.

## CONFLICT OF INTEREST

The authors declare no conflicts of interest.

### PEER REVIEW

The peer review history for this article is available at https://publons.com/publon/10.1002/brb3.2557

## Supporting information

SUPPORTING INFORMATION

## Data Availability

The data that support the findings of this study are available on request from the corresponding author. The data are not publicly available due to privacy or ethical restrictions.

## References

[brb32557-bib-0001] Abramowitz, J. S. , Deacon, B. J. , & Whiteside, S. P. H. (2011). Exposure therapy for anxiety: Principles and practice. Guilford Press.

[brb32557-bib-0002] Ashcraft, M. H. (2002). Math anxiety: Personal, educational, and cognitive consequences. Current Directions in Psychological Science, 11(5), 181–185. 10.1111/1467-8721.00196

[brb32557-bib-0003] Ashcraft, M. H. , & Kirk, E. P. (2001). The relationships among working memory, math anxiety, and performance. Journal of Experimental Psychology General, 130(2), 224–237. 11409101 10.1037//0096-3445.130.2.224

[brb32557-bib-0004] Ashcraft, M. H. , & Krause, J. A. (2007). Working memory, math performance, and math anxiety. Psychonomic Bulletin & Review, 14(2), 243–248. Retrieved from http://www.ncbi.nlm.nih.gov/pubmed/17694908 17694908 10.3758/bf03194059

[brb32557-bib-0005] Berch, D. B. (2005). Making sense of number sense: Implications for children with mathematical disabilities. Journal of Learning Disabilities, 38(4), 333–339. 10.1177/00222194050380040901 16122065

[brb32557-bib-0006] Biatchford, P. (1996). Pupils’ views on school work and school from 7 to 16 years. Research Papers in Education, 11(3), 263–288. 10.1080/0267152960110305

[brb32557-bib-0007] Butterworth, B. , & Laurillard, D. (2010). Low numeracy and dyscalculia: Identification and intervention. ZDM, 42(6), 527–539. 10.1007/s11858-010-0267-4

[brb32557-bib-0008] Butterworth, B. , Varma, S. , & Laurillard, D. (2011). Dyscalculia: From brain to education. Science, 332(6033), 1049–1053. 10.1126/science.1201536 21617068

[brb32557-bib-0009] Caviola, S. , Carey, E. , Mammarella, I. C. , & Szucs, D. (2017). Stress, time pressure, strategy selection and math anxiety in mathematics: A review of the literature. Frontiers in Psychology, 8, 1488. 10.3389/fpsyg.2017.01488 28919870 PMC5585192

[brb32557-bib-0010] Chang, T. T. , Lee, J. R. , & Yen, N. S. (2019). Mathematical learning and its difficulties in Taiwan: Insights from educational practice. In A. Fritz , V. G. Haase , & P. Räsänen (Eds.), International handbook of mathematical learning difficulties: From the laboratory to the classroom (pp. 265–278). Springer International Publishing.

[brb32557-bib-0011] Ching, B. H.‐H. (2017). Mathematics anxiety and working memory: Longitudinal associations with mathematical performance in Chinese children. Contemporary Educational Psychology, 51, 99–113. 10.1016/j.cedpsych.2017.06.006

[brb32557-bib-0012] Chiu, L.‐H. O. L. H. , & Henry, L. (1990). Development and validation of the Mathematics Anxiety Scale for Children. Measurement and Evaluation in Counseling and Development, 23, 121–127.

[brb32557-bib-0013] Choe, K. W. , Jenifer, J. B. , Rozek, C. S. , Berman, M. G. , & Beilock, S. L. (2019). Calculated avoidance: Math anxiety predicts math avoidance in effort‐based decision‐making. Science Advances, 5(11), eaay1062. 10.1126/sciadv.aay1062 31799398 PMC6867883

[brb32557-bib-0014] Craske, M. G. , Treanor, M. , Conway, C. C. , Zbozinek, T. , & Vervliet, B. (2014). Maximizing exposure therapy: An inhibitory learning approach. Behaviour Research and Therapy, 58, 10–23. 10.1016/j.brat.2014.04.006 24864005 PMC4114726

[brb32557-bib-0015] De Smedt, B. , Noël, M.‐P. , Gilmore, C. , & Ansari, D. (2013). How do symbolic and non‐symbolic numerical magnitude processing skills relate to individual differences in children's mathematical skills? A review of evidence from brain and behavior. Trends in Neuroscience and Education, 2(2), 48–55. 10.1016/j.tine.2013.06.001

[brb32557-bib-0016] Detterman, D. K. , & Daniel, M. H. (1989). Correlations of mental tests with each other and with cognitive variables are highest for low IQ groups. Intelligence, 13(4), 349–359. 10.1016/S0160-2896(89)80007-8

[brb32557-bib-0017] Engle, R. W. (2002). Working memory capacity as executive attention. Current Directions in Psychological Science, 11(1), 19–23. 10.1111/1467-8721.00160

[brb32557-bib-0018] Facon, B. (2006). Does age moderate the effect of IQ on the differentiation of cognitive abilities during childhood? Intelligence, 34(4), 375–386. 10.1016/j.intell.2005.12.003

[brb32557-bib-0019] Foley, A. E. , Herts, J. B. , Borgonovi, F. , Guerriero, S. , Levine, S. C. , & Beilock, S. L. (2017). The math anxiety‐performance link: A global phenomenon. Current Directions in Psychological Science, 26(1), 52–58.

[brb32557-bib-0020] Gunderson, E. , Park, D. , Maloney, E. , Beilock, S. , & Levine, S. (2017). Reciprocal relations among motivational frameworks, math anxiety, and math achievement in early elementary school. Journal of Cognition and Development, 19, 21–46. 10.1080/15248372.2017.1421538

[brb32557-bib-0021] Hembree, R. (1990). The nature, effects, and relief of mathematics anxiety. Journal for Research in Mathematics Education, 21(1), 33–46. 10.2307/749455

[brb32557-bib-0022] Hung, L.‐Y. , & Lian, W.‐H. (2015). Basic mathematical core skill test. Chinese Behavioral Science Corporation.

[brb32557-bib-0023] Jansen, B. R. J. , Louwerse, J. , Straatemeier, M. , Van der Ven, S. H. G. , Klinkenberg, S. , & Van der Maas, H. L. J. (2013). The influence of experiencing success in math on math anxiety, perceived math competence, and math performance. Learning and Individual Differences, 24, 190–197. 10.1016/j.lindif.2012.12.014

[brb32557-bib-0024] Kalmpourtzis, G. (2018). Educational game design fundamentals: A journey to creating intrinsically motivating learning experiences. CRC Press.

[brb32557-bib-0025] Kim, N. , Jang, S. , & Cho, S. (2018). Testing the efficacy of training basic numerical cognition and transfer effects to improvement in children's math ability. Frontiers in Psychology, 9, 1775. 10.3389/fpsyg.2018.01775 30333768 PMC6175973

[brb32557-bib-0026] Krinzinger, H. , Kaufmann, L. , & Willmes, K. (2009). Math anxiety and math ability in early primary school years. Journal of Psychoeducational Assessment, 27(3), 206–225. 10.1177/0734282908330583 20401159 PMC2853710

[brb32557-bib-0027] Lee, J. (2009). Universals and specifics of math self‐concept, math self‐efficacy, and math anxiety across 41 PISA 2003 participating countries. Learning and Individual Differences, 19(3), 355–365. 10.1016/j.lindif.2008.10.009

[brb32557-bib-0028] Ma, X. (1999). A meta‐analysis of the relationship between anxiety toward mathematics and achievement in mathematics. Journal for Research in Mathematics Education, 30(5), 520–540. 10.2307/749772

[brb32557-bib-0029] Maloney, E. A. , & Beilock, S. L. (2012). Math anxiety: Who has it, why it develops, and how to guard against it. Trends in Cognitive Sciences, 16(8), 404–406. 10.1016/j.tics.2012.06.008 22784928

[brb32557-bib-0030] Maloney, E. A. , Ramirez, G. , Gunderson, E. A. , Levine, S. C. , & Beilock, S. L. (2015). Intergenerational effects of parents' math anxiety on children's math achievement and anxiety. Psychological Science, 26(4), 1480–1488. 10.1177/0956797615592630 26253552

[brb32557-bib-0031] Maloney, E. A. , Risko, E. F. , Ansari, D. , & Fugelsang, J. (2010). Mathematics anxiety affects counting but not subitizing during visual enumeration. Cognition, 114(2), 293–297. 10.1016/j.cognition.2009.09.013 19896124

[brb32557-bib-0032] Mullis, I. V. S. , Martin, M. O. , Foy, P. , & Arora, A. (2012). TIMSS 2011 International Results in Mathematics. Retrieved from http://timssandpirls.bc.edu/timss2011/international‐results‐mathematics.html

[brb32557-bib-0033] Mullis, I. V. S. , Martin, M. O. , Foy, P. , & Hooper, M. (2016). TIMSS 2015 International Results in Mathematics. Retrieved from http://timssandpirls.bc.edu/timss2015/international‐results/

[brb32557-bib-0034] Nemmi, F. , Helander, E. , Helenius, O. , Almeida, R. , Hassler, M. , Rasanen, P. , & Klingberg, T. (2016). Behavior and neuroimaging at baseline predict individual response to combined mathematical and working memory training in children. Developmental Cognitive Neuroscience, 20, 43–51. 10.1016/j.dcn.2016.06.004 27399278 PMC6987694

[brb32557-bib-0035] Noguchi, K. , Gel, Y. R. , Brunner, E. , & Konietschke, F. (2012). nparLD: An R software package for the nonparametric analysis of longitudinal data in factorial experiments. Journal of Statistical Software, 50(12), 1–23. 10.18637/jss.v050.i12 25317082

[brb32557-bib-0036] Obersteiner, A. , Reiss, K. , & Ufer, S. (2013). How training on exact or approximate mental representations of number can enhance first‐grade students’ basic number processing and arithmetic skills. Learning and Instruction, 23, 125–135. 10.1016/j.learninstruc.2012.08.004

[brb32557-bib-0037] OECD . (2013). PISA 2012 Results: Ready to learn (Vol. III).

[brb32557-bib-0038] OECD . (2019). PISA 2018 Results (Volume III): What school life means for students’ lives.

[brb32557-bib-0039] Park, D. , Ramirez, G. , & Beilock, S. L. (2014). The role of expressive writing in math anxiety. Journal of Experimental Psychology‐Applied, 20(2), 103–111. 10.1037/xap0000013 24708352

[brb32557-bib-0040] Passolunghi, M. C. (2011). Cognitive and emotional factors in children with mathematical learning disabilities. International Journal of Disability, Development and Education, 58(1), 61–73. 10.1080/1034912X.2011.547351

[brb32557-bib-0041] Plass, J. L. , Homer, B. D. , & Kinzer, C. K. (2015). Foundations of game‐based learning. Educational Psychologist, 50(4), 258–283. 10.1080/00461520.2015.1122533

[brb32557-bib-0042] Ramirez, G. , Gunderson, E. A. , Levine, S. C. , & Beilock, S. L. (2013). Math anxiety, working memory, and math achievement in early elementary school. Journal of Cognition and Development, 14(2), 187–202. 10.1080/15248372.2012.664593

[brb32557-bib-0043] Ramirez, G. , Shaw, S. T. , & Maloney, E. A. (2018). Math anxiety: Past research, promising interventions, and a new interpretation framework. Educational Psychologist, 53(3), 145–164. 10.1080/00461520.2018.1447384

[brb32557-bib-0044] Räsänen, P. , Salminen, J. , Wilson, A. J. , Aunio, P. , & Dehaene, S. (2009). Computer‐assisted intervention for children with low numeracy skills. Cognitive Development, 24(4), 450–472. 10.1016/j.cogdev.2009.09.003

[brb32557-bib-0045] Rattan, A. , Good, C. , & Dweck, C. S. (2012). “It's ok – Not everyone can be good at math”: Instructors with an entity theory comfort (and demotivate) students. Journal of Experimental Social Psychology, 48(3), 731–737. 10.1016/j.jesp.2011.12.012

[brb32557-bib-0046] Sanchez‐Perez, N. , Castillo, A. , Lopez‐Lopez, J. A. , Pina, V. , Puga, J. L. , Campoy, G. , García‐Santos, J. M. , González‐Salinas, C. , & Fuentes, L. J. (2017). Computer‐based training in math and working memory improves cognitive skills and academic achievement in primary school children: Behavioral results. Frontiers in Psychology, 8, 2327. 10.3389/fpsyg.2017.02327 29375442 PMC5767320

[brb32557-bib-0047] Suárez‐Pellicioni, M. , Núñez‐Peña, M. I. , & Colomé, À. (2016). Math anxiety: A review of its cognitive consequences, psychophysiological correlates, and brain bases. Cognitive, Affective, & Behavioral Neuroscience, 16(1), 3–22. 10.3758/s13415-015-0370-7 26250692

[brb32557-bib-0048] Suinn, R. M. , Taylor, S. , & Edwards, R. W. (1988). Suinn mathematics anxiety rating‐scale for elementary‐school students (Mars‐E)—Psychometric and normative data. Educational and Psychological Measurement, 48(4), 979–986. 10.1177/0013164488484013

[brb32557-bib-0049] Sun, Y. , & Pzydrowski, L. (2009). Using technology as a tool to reduce mathematics anxiety. The Journal of Human Resource and Adult Learning, 5(2), 38–44.

[brb32557-bib-0050] Supekar, K. , Iuculano, T. , Chen, L. , & Menon, V. (2015). Remediation of childhood math anxiety and associated neural circuits through cognitive tutoring. Journal of Neuroscience, 35(36), 12574–12583. 10.1523/JNEUROSCI.0786-15.2015 26354922 PMC4563039

[brb32557-bib-0051] Szűcs, D. , & Myers, T. (2017). A critical analysis of design, facts, bias and inference in the approximate number system training literature: A systematic review. Trends in Neuroscience and Education, 6, 187–203. 10.1016/j.tine.2016.11.002

[brb32557-bib-0052] Tan, J. B. , & Yates, S. (2011). Academic expectations as sources of stress in Asian students. Social Psychology of Education, 14(3), 389–407. 10.1007/s11218-010-9146-7

[brb32557-bib-0053] Vanbecelaere, S. , Van den Berghe, K. , Cornillie, F. , Sasanguie, D. , Reynvoet, B. , & Depaepe, F. (2020). The effects of two digital educational games on cognitive and non‐cognitive math and reading outcomes. Computers & Education, 143, 103680. 10.1016/j.compedu.2019.103680

[brb32557-bib-0054] Van Etten, M. L. , & Taylor, S. (1998). Comparative efficacy of treatments for post‐traumatic stress disorder: A meta‐analysis. Clinical Psychology & Psychotherapy, 5(3), 126–144. 10.1002/(Sici)1099-0879(199809)5:3<126::Aid‐Cpp153>3.0.Co;2‐H

[brb32557-bib-0055] Verkijika, S. F. , & De Wet, L. (2015). Using a brain‐computer interface (BCI) in reducing math anxiety: Evidence from South Africa. Computers & Education, 81, 113–122. 10.1016/j.compedu.2014.10.002

[brb32557-bib-0056] Wechsler, D. (2004). The Wechsler intelligence scale for children (4th ed.). Pearson Assessment.

[brb32557-bib-0057] Wilson, A. J. , Dehaene, S. , Pinel, P. , Revkin, S. K. , Cohen, L. , & Cohen, D. (2006). Principles underlying the design of “The Number Race,” an adaptive computer game for remediation of dyscalculia. Behavioral and Brain Functions, 2, 19. 10.1186/1744-9081-2-19 16734905 PMC1550244

[brb32557-bib-0058] Wilson, A. J. , Revkin, S. K. , Cohen, D. , Cohen, L. , & Dehaene, S. (2006). An open trial assessment of “The Number Race,” an adaptive computer game for remediation of dyscalculia. Behavioral and Brain Functions, 2, 20. 10.1186/1744-9081-2-20 16734906 PMC1523349

[brb32557-bib-0059] Wolitzky‐Taylor, K. B. , Horowitz, J. D. , Powers, M. B. , & Telch, M. J. (2008). Psychological approaches in the treatment of specific phobias: A meta‐analysis. Clinical Psychology Review, 28(6), 1021–1037. 10.1016/j.cpr.2008.02.007 18410984

[brb32557-bib-0060] Wu, S. S. , Barth, M. , Amin, H. , Malcarne, V. , & Menon, V. (2012). Math anxiety in second and third graders and its relation to mathematics achievement. Frontiers in Psychology, 3, 162. 10.3389/fpsyg.2012.00162 22701105 PMC3369194

[brb32557-bib-0061] Young, C. B. , Wu, S. S. , & Menon, V. (2012). The neurodevelopmental basis of math anxiety. Psychological Science, 23(5), 492–501. 10.1177/0956797611429134 22434239 PMC3462591

[brb32557-bib-0062] Zhang, J. , Zhao, N. , & Kong, Q. P. (2019). The relationship between math anxiety and math performance: A meta‐analytic investigation. Frontiers in Psychology, 10, 1613–1613. 10.3389/fpsyg.2019.01613 31447719 PMC6692457

