# Supplementary material for: Evaluation of math anxiety and its remediation through a digital training program in mathematics for first and second graders
Source: Brain Behav. 2022 Mar 29;12(5):e2557. doi: 10.1002/brb3.2557 (PMC9120910; doi:10.1002/brb3.2557)
Supplement: Supplementary file 1 — SUPPORTING INFORMATION [file BRB3-12-e2557-s001.docx]

# Supporting Information

**Table S1**

*Results of nonparametric analysis on the differences in math anxiety for the low-intensity training group*

| Effect | *df* | $F$*/*$\chi^{2}$ | *p* |
| --- | --- | --- | --- |
| Low-intensity training group | | | |
| Initial math anxiety | 1 | 58.47 | <.001 |
| Time | 1 | 7.43 | .006 |
| Initial math anxiety × Time | 1 | 5.77 | .016 |
| Initial math anxiety = high | | | |
| Time | 1 | 0.51 | .474 |
| Initial math anxiety = low | | | |
| Time | 1 | 7.64 | .006 |
| Time = post-training | | | |
| Initial math anxiety | 1 | 12.8 | <.001 |

**Figure S1**

*Relations between initial math anxiety (MA) and the math anxiety changes in the low-intensity training group*


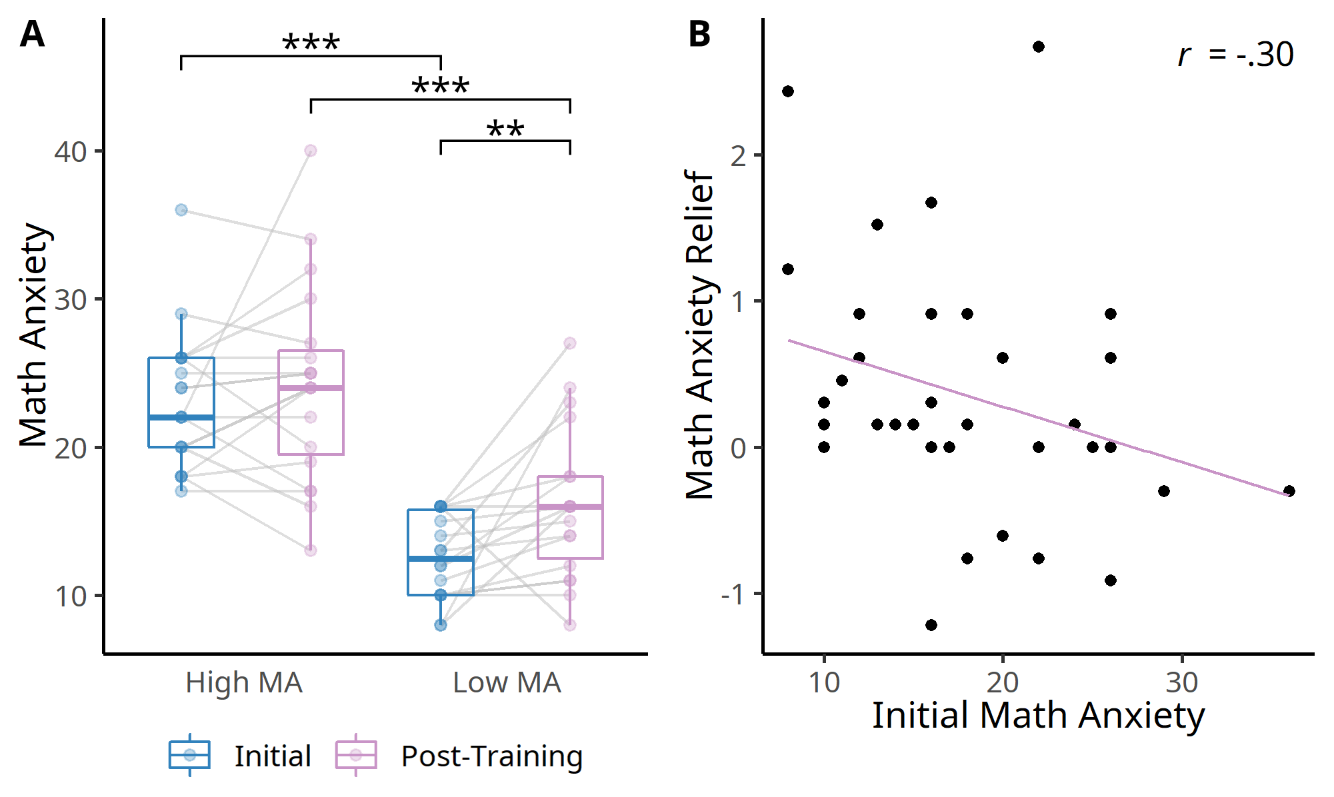


*Note.* (A) There was an interaction between time (Initial vs. Post-Training) and group (High vs. Low MA at baseline): Children with low MA showed increased math anxiety, and those with high MA remained highly math-anxious. (B) The initial math anxiety was not significantly associated with the math anxiety relief.

****p* < .001, ***p* < .01.
